# Reconstructing the Dissemination Dynamics of the Major HIV-1 Subtype B Non-Pandemic Lineage Circulating in Brazil

**DOI:** 10.3390/v11100909

**Published:** 2019-10-01

**Authors:** Ighor Arantes, Myuki Alfaia Esashika Crispim, Mônica Nogueira da Guarda Reis, Mariane Martins Araújo Stefani, Gonzalo Bello

**Affiliations:** 1 Laboratório de AIDS e Imunologia Molecular, Instituto Oswaldo Cruz, FIOCRUZ, Rio de Janeiro 21040-360, Brazil; 2 Fundação de Hematologia e Hemoterapia do Amazonas/HEMOAM, Manaus 69050-001, Brazil; 3 Instituto de Patologia Tropical e Saúde Pública, Universidade Federal de Goiás, Goiânia 74605-450, Brazil

**Keywords:** HIV-1, subtype B, non-pandemic, Brazil, Caribbean, phylogeography, phylodynamics

## Abstract

Non-pandemic variants of the Human Immunodeficiency Virus Type 1 (HIV-1) subtype B accounts for a significant fraction of HIV infections in several Caribbean islands, Northeastern South American countries and the Northern Brazilian states of Roraima and Amazonas. In this paper, we used a comprehensive dataset of HIV-1 subtype B *pol* sequences sampled in Amazonas and Roraima between 2007 and 2017 to reconstruct the phylogeographic and demographic dynamics of the major HIV-1 subtype B non-pandemic Brazilian lineage, designated as B_CAR-BR-I_. Our analyses revealed that its origin could be traced to one of many viral introductions from French Guiana and Guyana into Northern Brazil, which probably occurred in the state of Amazonas around the late 1970s. The B_CAR-BR-I_ clade was rapidly disseminated from Amazonas to Roraima, and the epidemic grew exponentially in these Northern Brazilian states during the 1980s and 1990s, coinciding with a period of economic and fast population growth in the region. The spreading rate of the B_CAR-BR-I_ clade, however, seems to have slowed down since the early 2000s, despite the continued expansion of the HIV-1 epidemic in this region in the last decade.

## 1. Introduction

According to the last report of the Brazilian Ministry of Health, the number of new Human Immunodeficiency Virus Type 1 (HIV-1) infections remained roughly stable or decreased in most Brazilian states from 2007 to 2017 [1]. In the Northern Brazilian region, by contrast, the HIV epidemic expanded continuously and notable increases in the AIDS incidence rate were recorded in the last decade in the states of Roraima (28%), Amazonas (35%), Para (55%), Amapá (68%) and Tocantins (143%) [1]. In the 2018 AIDS incidence rate ranking of all Brazilian states, Roraima and Amazonas respectively occupyied the first and fourth positions [1].

The HIV-1 subtype B spread in the Americas from a founder strain probably introduced in the island of Hispaniola (shared by Haiti and the Dominican Republic) around the mid-1960s [2,3,4]. Most current subtype B infections are driven by the dissemination of a pandemic clade (B_PANDEMIC_) that spread worldwide from North America [2,3,4]; however, other ancestral non-pandemic subtype B variants (B_CAR_) are also detected in the Caribbean region [2,3,4]. Dispersed initially from the island of Hispaniola, the B_CAR_ strains subsequently originated local clades in Trinidad and Tobago (B_CAR-TT_) and Jamaica in the early 70s [2,3,4]. From Trinidad and Tobago, the B_CAR-TT_ lineage landed in Northern South America in the mid-1970s, producing the most prevalent subtype B non-pandemic clade (B_CAR-SA-I_) circulating in French Guiana, Guyana, Suriname and Brazil [5].

Multiple B_CAR_ strains were introduced into Brazil, but only some of them established onward transmissions and local clades [5,6]. The major non-pandemic subtype B Brazilian clade previously identified, named B_CAR-BR-I_, is a tributary of the B_CAR-SA-I_ clade [5] and accounts for an important fraction of HIV-1 subtype B infections in Roraima (~33%) and Amazonas (~15%) [6,7]. Previous analyses support that the B_CAR-BR-I_ clade probably arose by the dissemination of a founder B_CAR-SA-I_ strain from French Guiana into Roraima around the late 1970s [5,6]. Those studies, however, were based on a limited number of B_CAR-BR-I_ sequences mostly (63%) sampled in Roraima, which may have introduced a significant sampling bias on phylogeographic reconstructions. Furthermore, the precise demographic dynamics of this major non-pandemic Brazilian lineage remains unknown.

The recent characterization of new subtype B non-pandemic sequences from Amazonas state [7] allowed us to compile a more comprehensive dataset of 76 HIV-1 B_CAR-BR-I_ *pol* sequences from Amazonas (*n* = 45), Roraima (*n* = 29), Rondonia (*n* = 1) and Sao Paulo (*n* = 1), collected between 2007 and 2017. With this new dataset, we obtain a more accurate reconstruction of the spatiotemporal origin of the HIV-1 B_CAR-BR-I_ clade in Northern Brazil and we also infer the demographic dynamics of this lineage for the first time.

## 2. Materials and Methods

### 2.1. HIV-1 B_CAR_ pol Sequence Dataset

A total of 76 HIV-1 subtype B *pol* sequences from Brazil that covered the entire protease and partial reverse transcriptase (PR/RT) regions (nucleotides 2253–3260 relative to HXB2 clone) and were previously classified within the B_CAR-BR-I_ clade [6,7] were used in the present study (Table 1). HIV-1 Brazilian sequences were aligned with subtype B_CAR_ *pol* sequences from Hispaniola (*n* = 130), noted as the most probable epicenter of subtype B epidemic, and with B_CAR_ *pol* sequences representative of the B_CAR-TT_ (*n* = 41) and B_CAR-SA-I_ clades (*n* = 69) circulating in Trinidad and Tobago, French Guiana, Guyana and Suriname, that were described as the non-Brazilian lineages most closely related to the B_CAR-BR-I_ clade [3,5,6] (see Table S1 for details about GenBank accession number, geographic origin and sampling time of all HIV-1 B_CAR_ sequences used in this study). Subtype D *pol* sequences from the Democratic Republic of Congo (DRC) (*n* = 10), noted as the most probable source location of subtype B strain introduced in the Americas [2], were used as outgroup. Sequences were aligned using the Clustal W program [8], and all sites associated with major antiretroviral drug resistance in PR and RT were excluded. The presence of putative intra-subtype recombinant sequences among the subtype B datasets was analyzed using the RDP4 software [9], with those sequences selected as such by three or more of the algorithms being deemed as recombinant.

### 2.2. Evolutionary, Phylogeographic, and Demographic Analyses

The evolutionary rate, the age of the most recent common ancestor (*T*_MRCA_), the spatial diffusion pattern and the rate of population growth (r, years^−1^) of HIV-1 B_CAR-BR-I_ clade circulating in Brazil were jointly estimated using the Bayesian Markov Chain Monte Carlo (MCMC) approach as implemented in BEAST v1.10 [10,11] with BEAGLE [12] to improve run-time. Analyses were performed using the GTR + I + Г_4_ nucleotide substitution model and a relaxed uncorrelated lognormal molecular clock model [13]. Inspection of temporal structure with program TempEst [14] revealed that the B_CAR_ *pol* dataset compiled here did not contain sufficient temporal signal for reliable time-scale estimations (X-intercept (TMRCA) < 1910). Thus, Bayesian MCMC analyses were performed using a normal prior distribution on the substitution rate (mean = 2.1 × 10^−3^ substitution/site/year, standard deviation = 1.0 × 10^−4^) based on previous estimates for the subtype B *pol* gene [15,16,17,18]. Migration events were reconstructed using a reversible discrete phylogeographic model [19] with a CTMC rate reference prior [20]. Changes in effective population size through time (Ne) were estimated using the non-parametric Bayesian Skygrid (BSKG) model [21]. Estimates of the *r* were obtained under the best fit parametric model selected using the log marginal likelihood estimation (MLE) based on the generalized stepping-stone sampling (GSS) method [22]. The mean basic reproductive number (R_0_) was inferred with the formula R_0_ = *r*D + 1, where *r*, the epidemic growth rate, derived from the best fit parametric model, and D, the average duration of the infection, was considered as eight years. Three MCMC chains were run for 200 × 10^6^ generations and then combined using LogCombiner v1.10. Convergence and uncertainty of parameter estimates were assessed by calculating the Effective Sample Size (ESS) and 95% Highest Probability Density (HPD) values, respectively, after excluding the initial 10% of each run with Tracer v1.7 [23]. Convergence of parameters was considered with ESS ≥ 200. The maximum clade credibility (MCC) tree was summarized with TreeAnnotator v1.10 and visualized with FigTree v1.4.4 [24].

## 3. Results

### 3.1. Dispersal Pattern of the HIV-1 B_CAR_ Strains from the Caribbean to Brazil

**Figure 1:**
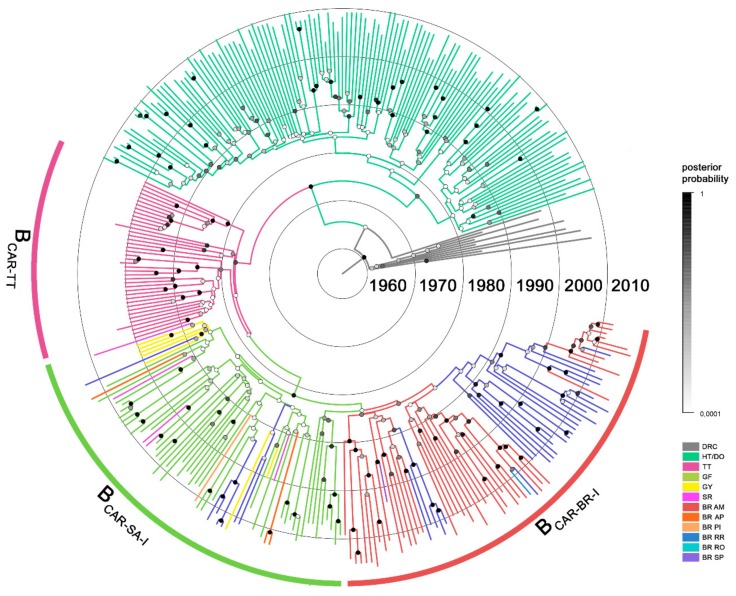
Time-scaled Bayesian maximum clade credibility (MCC) tree of HIV-1 B_CAR_ *pol* sequences from Brazil (*n* = 87), Northern South American countries (*n* = 58), and the Caribbean (*n* = 171) and subtype D reference sequences (*n* = 10) from the Democratic Republic of Congo. Branches are colored according to the most probable location state of their descendent nodes as indicated in the legend at right. Posterior clade probability for all nodes is indicated in the color scale on the right. Branch lengths are depicted in units of time (years). The circular brackets highlight the position of major non-pandemic HIV-1 B_CAR_ clades circulating in Trinidad and Tobago (B_CAR-TT_), Northern South America (B_CAR-SA-I_) and Brazil (B_CAR-BR-I_). The tree was automatically rooted under the assumption of a relaxed molecular clock. DRC: Democratic Republic of Congo; HT/DO: Haiti/Dominican Republic; TT: Trinidad and Tobago; GF: French Guiana; GY: Guyana; SR: Suriname; BR: Brazil; AM: Amazonas; AP: Amapá; PI: Piauí; RR: Roraima; RO: Rondônia; SP: São Paulo.

**Figure 2:**
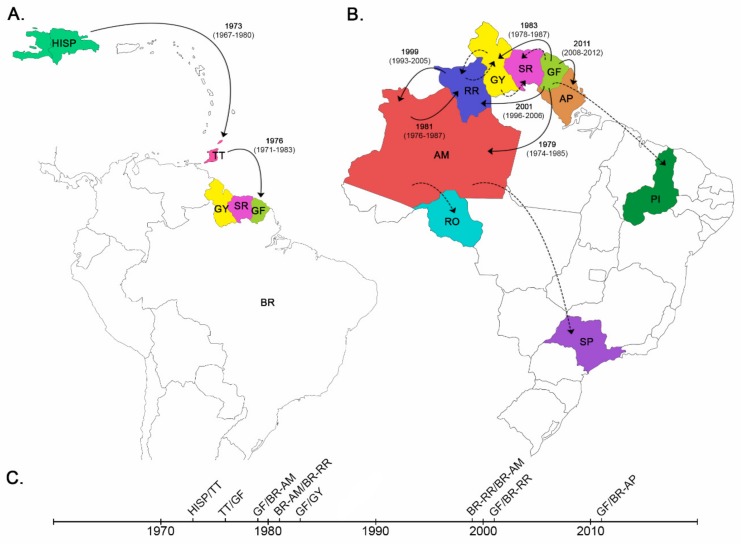
Spatiotemporal dynamics of the dissemination of non-pandemic HIV-1 B_CAR_ clades in the Caribbean and South American regions. (**A**,**B**) Lines between locations represent branches in the Bayesian MCC tree along which location transitions occurred. Migration events that originated secondary outbreaks or sporadic infections with no evidence of subsequent dispersion are represented by solid and dashed lines, respectively. The median T_MRCA_ (and 95% HPD interval) is indicated for the earliest migration events between locations that originated secondary outbreaks. All migration events displayed *PSP* support ≥ 0.55 and nearly all T_MRCA_ estimates (with the exception of the earliest migration from AM to RR) were estimated from local sub-clades with high support (PP ≥ 0.75). (**C**) Timeline summarizing the main migratory events. AM: Amazonas; AP: Amapá; GF: French Guiana; GY: Guyana; HISP: Hispaniola; PI: Piauí; RO: Rondônia; RR: Roraima; SP: São Paulo; SR: Suriname; TT: Trinidad and Tobago. Maps were created from templates obtained from d-maps.com [25].

HIV-1 subtype B non-pandemic *pol* sequences from Brazil, Northern South America and Trinidad and Tobago previously classified as B_CAR-BR-I_, B_CAR-SA-I_ and B_CAR-TT_ were combined with B_CAR_ sequences from Hispaniola (Table 1) and subsequently subjected to Bayesian phylogeographic reconstructions. The mean estimated evolutionary rate of the HIV-1 B_CAR_/D *pol* dataset was 2.0 × 10^-3^ substitutions/site/year (95% HPD 2.0 × 10^−3^–2.2 × 10^−3^ substitutions/site per year), whereas the corresponding median coefficient of rate variation was 0.28 (95% HPD: 0.24–0.34), supporting the selection of a relaxed molecular clock model. Consistent with previous findings [2,3], the origin of the well-supported sub-clades B_CAR-TT_ (*posterior probability* (*PP*) = 0.76) and B_CAR-SA-I_ (*PP* = 0.93) was traced to the sequential viral movement from Hispaniola to Trinidad and Tobago (*posterior state probability* (*PSP*) = 0.59) around the early 1970s and from Trinidad and Tobago to French Guiana (*PSP* = 0.85) around the mid-1970s, respectively (Figure 1 and Figure 2A, Table 2).

The B_CAR-SA-I_ lineage rapidly spread across Northern South American countries (French Guiana, Guyana and Suriname) during the 1970s and was then independently disseminated from French Guiana (PSP ≥ 0.97) and Guyana (PSP = 0.98) into Northern Brazil (Amazonas, Amapá and Roraima) at multiple times (Figure 1, Figure 2B). One B_CAR-SA-I_ strain introduced from French Guiana originates the sub-lineage B_CAR-BR-I_ that was successfully disseminated in Amazonas and Roraima. It is interesting to note that the *PP* support of the lineage B_CAR-BR-I_ increased from 0.56 to 0.85 when Brazilian B_CAR-SA-I_ strains branching outside the major Brazilian clade were removed from analysis. This suggests that some Brazilian B_CAR_ strains may represent mosaic forms between different B_CAR-SA-I_ sub-lineages, despite none of the B_CAR_ sequences included in our analyses been identified as intra-subtype recombinants. Others B_CAR-SA-I_ strains introduced from French Guiana or Guyana in Northern Brazil gave origin to small sub-clades (n ≤ 4) in Roraima (*PP* = 1) and Amapa (*PP* = 1), or resulted in sporadic infections with no evidence of subsequent dispersion.

### 3.2. Dissemination of the HIV-1 B_CAR-BR-I_ Lineage in Northern Brazil

According to our phylogeographic reconstruction, the B_CAR-BR-I_ lineage was most probably introduced in the state of Amazonas (*PSP* = 0.92) around the late 1970s and was then repeatedly disseminated between Amazonas and Roraima (Figure 1 and Figure 2B). The number of B_CAR-BR-I_ strains from Amazonas (*n* = 45) included in our dataset was higher than the corresponding number of sequences from Roraima (*n* = 29), and this sampling bias might confound the Bayesian phylogeographic reconstructions. To test this hypothesis, we constructed two sub-datasets, each randomly containing half of the sequences from Amazonas. When subjected to Bayesian phylogeographic reconstructions, the root location for the B_CAR-BR-I_ clade remained in the state of Amazonas as the most probable hypothesis (*PSP* > 0.90, Table 3).

The phylogeographic analysis supports at least four independent disseminations of the B_CAR-BR-I_ lineage from Amazonas (*PSP* ≥ 0.79) into Roraima. The earliest viral introduction into Roraima was traced to around the early 1980s (Figure 2B), but that migration event was inferred from a large Roraima’s sub-clade with low support (*PP* = 0.17) and should thus be interpreted with caution. Smaller sub-clades from Roraima with high support (*PP* > 0.70) point to the local circulation of the B_CAR-BR-I_ lineage in this Northern state from the mid-1980s onwards. Our analysis also reveals at least two independent viral introductions from Roraima (*PSP* ≥ 0.97) into Amazonas with the subsequent dissemination of one of them, giving origin to a well-supported (*PP* = 1) Amazonian sub-clade around the late 1990s (Figure 1 and Figure 2B). Sporadic disseminations the B_CAR-BR-I_ lineage from Amazonas to Rondonia (*PSP* = 0.79) and Sao Paulo (*PSP* = 1) were also detected, but with no evidence of further local transmission.

**Figure 3:**
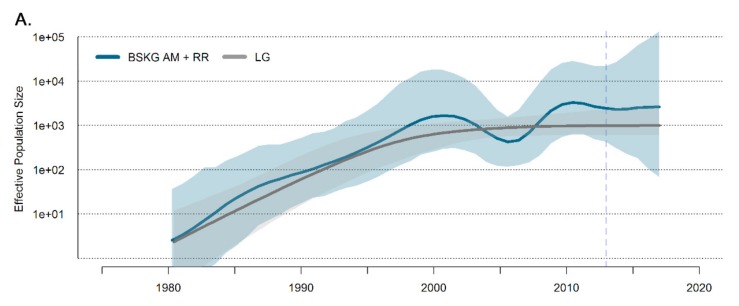

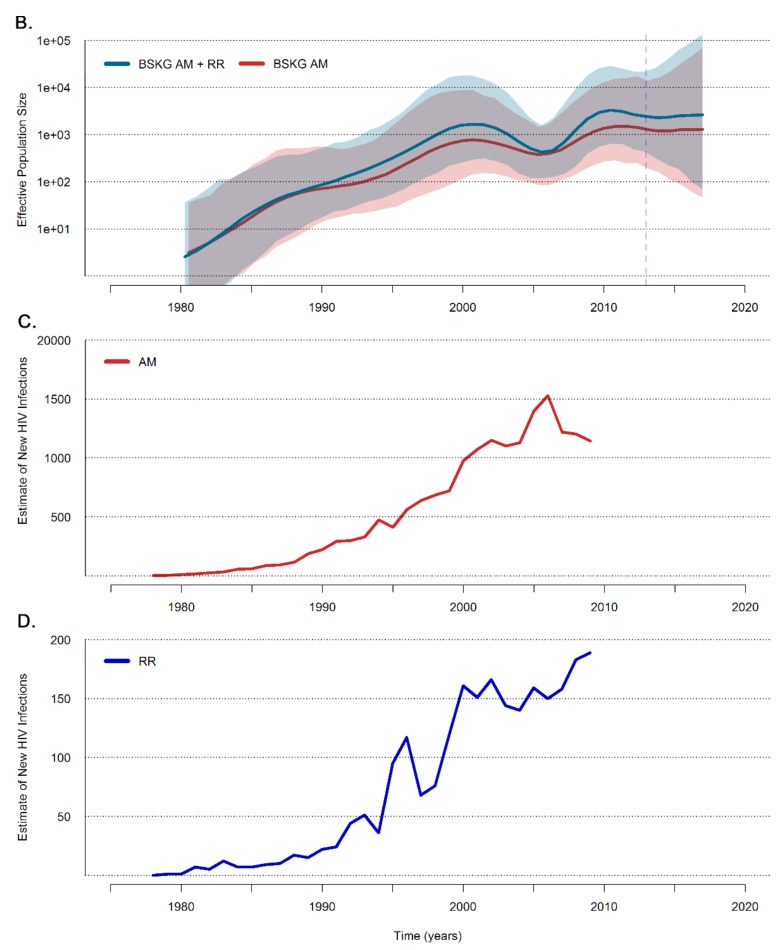
Demographic history of the HIV-1 B_CAR-BR-I_ clade. (**A**,**B**) Median estimate of the effective population size (N_e_) for datasets comprising B_CAR-BR-I_ sequences from Roraima and Amazonas (blue line) and from Amazonas only (red line) using the Bayesian Skygrid (BSKG) model along with their 95% highest probability density (HPD) intervals (pale blue and pale red areas). The median N_e_ estimates provided by the logistic coalescent-based parametric model (LG; dark gray line) and its 95% HPD (pale gray area) are co-plotted in the first graphic. The vertical blue dashed lines indicate the time of the last coalescent event. (**C**,**D**) Number of new HIV infections among adults (15+) in Amazonas and Roraima states estimated by subtracting eight years from new AIDS cases notified between 1986 and 2017 [26].

Given that the lineage B_CAR-BR-I_ might present different dynamics in each Brazilian state, we reconstruct the demographic history of this lineage with the BSKG model using sequences from both Amazonas and Roraima states (Figure 3A) and only from Amazonas state (Figure 3B). The evolution of the effective population size (N_e_) was quite similar in both analyses and supports a sustained exponential growth phase until the early 2000s, followed by a transient period of epidemic stabilization until the mid-2000s, a resumed growth until the late 2000s and a new stabilization phase that extends until the last coalescent event. These results displayed some differences with the temporal trends of the estimated number of new HIV cases in Amazonas (Figure 3C), but roughly matched with those described in Roraima (Figure 3D). According to the epidemiological data, the number of new HIV cases in Amazonas grew continuously until 2005 and then decreased until 2009, while the epidemic in Roraima grew until the early 2000s, remained roughly stable until the mid-2000s and then resumed growth.

**Figure 4:**
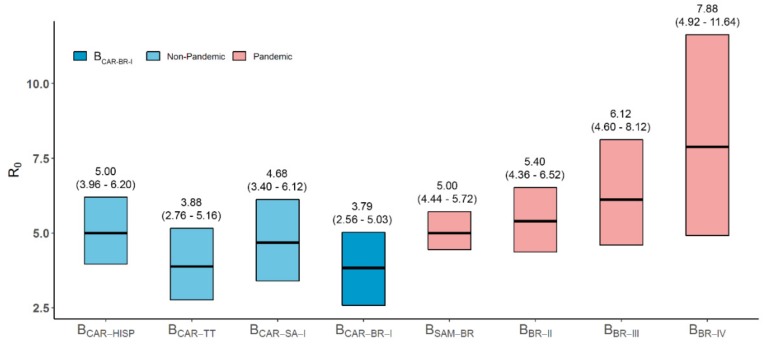
Coalescent-based estimates of the basic reproductive number (R_0_) of clade B_CAR-BR-I_ and other major pandemic and non-pandemic HIV-1 subtype B clades circulating in Brazil, Northern South America and the Caribbean. The boxes and the numbers above illustrate the median R_0_ and the 95% HPD intervals of the posterior distributions estimated under the logistic growth coalescent model for the HIV-1 B_CAR-BR-I_ clade (blue), related Caribbean B_CAR_ clades (light blue) and major Brazilian B_PANDEMIC_ clades (light red). R_0_ values for clades other than B_CAR-BR-I_ were obtained from previous studies [3,5,27].

Despite none of the simple parametric coalescent models being able to describe the intricate demographic pattern of the B_CAR-BR-I_ epidemic, the growth rate of the B_CAR-BR-I_ epidemic in Amazonas and Roraima during the first decades was estimated from the logistic growth model (Figure 3A), which was strongly supported over the exponential and expansion models (log BF > 3) (Supplementary Materials Table S2). According to the logistic model, the median initial growth rate of the B_CAR-BR-I_ epidemic was 0.35 (95% HPD: 0.19–0.50), which corresponds to a basic reproductive number (R_0_) of 3.79 (95% HPD: 2.56–5.03) (Figure 4).

## 4. Discussion

Our phylogeographic analysis revealed multiple introductions of the non-pandemic B_CAR-SA-I_ lineage into the Northern Brazilian states of Amazonas, Amapá and Roraima from neighboring countries (French Guiana and Guyana). Most of those introductions resulted in local transmission chains of small size (< 5 sequences) or dead-end infections, with the only exception of the founder event that originated the most prevalent non-pandemic HIV-1 subtype B Brazilian clade, designated as B_CAR-BR-I_. These results support that international migration along the Amazonian frontier represents an important driving force for recurrent introductions of non-pandemic subtype B variants into Northern Brazil, although the successful establishment of these variants in the country seems to be a rarer phenomenon.

Our study supports that the B_CAR-BR-I_ clade was the product of a successful transmission chain probably initiated in the Brazilian state of Amazonas around the late 1970s and later disseminated into Roraima. In a previous study conducted by our group, we traced the origin of the B_CAR-BR-I_ clade to the state of Roraima around the late 1970s [6]. In that work, nonetheless, we had to deal with the limitation of analyzing a much larger number of B_CAR_ sequences from Roraima (*n* = 32) than from Amazonas (*n* =14), notwithstanding the aforementioned state having, in 2018, a population of HIV-infected individuals over six times larger than Roraima [1]. The current finding that traced the origin of the B_CAR-BR-I_ clade in Amazonas was robust enough to sampling bias as this location was noted as the most probable geographic origin even when phylogeographic reconstructions were conducted with balanced datasets containing a roughly similar number of sequences from both Amazonas and Roraima states.

The origin of the B_CAR-BR-I_ clade in the Amazonas state around the late 1970s is in agreement with available epidemiological and historical information. The first reported AIDS cases in Amazonas dates back to 1986 [1], consistent with the circulation of HIV in the region since the late 1970s. Amazonas has been an important state of transit for Brazilian migrants en route to/from Guyana and Suriname [28] and by 1980, the Amazonas state had a much larger population (~1.5 million people) than Roraima (~82,000 million people), creating a more fertile ground for the establishment of non-pandemic subtype B strains introduced from neighboring Amazonian countries. The recurrent dissemination of B_CAR-BR-I_ strains between Amazonas and Roraima since the early 1980s onwards is also consistent with the intense human flux between both Brazilian states since the inauguration in 1977 of the BR-174 road connecting the state’s capitals [29]. The poor connectivity of Amazonas and Roraima to other Brazilian states might also explain the paucity of B_CAR-BR-I_ strains outside that region.

The population dynamics herein reconstructed for the HIV-1 B_CAR-BR-I_ epidemic in Amazonas and Roraima support an initial phase of exponential growth during the 1980s and 1990s. This exponential growth phase coincides with a constant increase in the number of new HIV cases in the states of Amazonas and Roraima (Figure 3) and also coincides with a significant population (Figure S1) and economic growth driven by the expansion of the local industrial park in Manaus (capital of Amazonas) and the rise of legal/illegal mining activities in Roraima [30,31,32]. Between 1980 and 2000, the population grew from 1.4 million to over 2.8 million individuals in Amazonas and from 82,000 to nearly 325,000 inhabitants in Roraima (Figure S1). These drastic changes in the demographic and economic scenarios certainly create an opportunity for the successful dissemination of HIV.

Demographic reconstruction supports a short phase of stabilization for the HIV-1 B_CAR-BR-I_ epidemic between the early and the mid-2000s, and a resumed period of growth until 2010. This pattern is quite consistent with the temporal changes in the estimated number of new HIV cases in Roraima but differs from the dynamic in Amazonas, where the estimated number of new HIV cases reached a peak in 2005 and declined until 2009. The exclusion of HIV sequences from Roraima imposed only minimal changes to the demographic reconstruction, indicating that the population dynamic of the B_CAR-BR-I_ epidemic might have followed a different trend than the overall HIV epidemic in the Amazonas state during the 2000s. It is important to note that because the B_CAR-BR-I_ clade comprises only a fraction of HIV-1 infections in Amazonas (<14%), its demographic dynamic may be different from other prevalent HIV-1 clades circulating in that Brazilian state. Our demographic reconstruction also supports the recent stabilization of the B_CAR-BR-I_ epidemic before the last coalescent event in 2013.

The median R_0_ here estimated for the B_CAR-BR-I_ epidemic (3.84) over the first two decades was roughly equivalent to those previously reported for the related B_CAR-TT_ (3.88) and B_CAR-SA-I_ (4.68) clades [3,5], but slightly lower than those described for major B_PANDEMIC_ Brazilian lineages (5.00–7.88) [27] (Figure 4). Although these results must be interpreted with prudence since they exhibit broad and overlapping 95% HPD intervals, the apparent differences in R_0_ values might reflect large transmissibility of B_PANDEMIC_ compared with B_CAR_ viruses circulating in Brazil or could expose discrepancies in the size and/or connectivity of the underlying transmission networks across different Brazilian regions. Future analyses comparing the R_0_ values of HIV-1 B_PANDEMIC_ and B_CAR_ transmission clusters circulating in Amazonas and Roraima will be of paramount importance to determine the relative impact of viral and/or transmission chain characteristics on the epidemic potential of different viral subtype B lineages.

Despite the fact that the B_CAR-BR-I_ dataset used in the present work comprises a more geographically balanced dataset than previous studies and that it could be useful for the spatial reconstruction of the lineage’s root, it is feasible that quite a larger number of B_CAR-BR-I_ sequences from Amazonas and Roraima states would be required for understanding the structure and dynamics of the B_CAR-BR-I_ transmission networks in those Northern Brazilian states. Assuming that the B_CAR-BR-I_ infections accounts for approximately 15 and 30% of HIV-1 subtype B infections in Amazonas and Roraima [6,7], respectively, our sampling density probably corresponds to approximately 2–5% of the B_CAR-BR-I_-infected individuals living in those Brazilian states. Such a sampling density is below the minimal level of 10% suggested by simulation studies for HIV-1 clusters analysis [33].

In summary, this study demonstrates that non-pandemic HIV-1 subtype B strains have been introduced at multiple times from the Northeastern South American region into Northern Brazil. One of these introductions that probably occurred in the state of Amazonas around the late 1970s gave origin to the most prevalent Brazilian B_CAR-BR-I_ clade. The B_CAR-BR-I_ clade was rapidly disseminated from Amazonas to Roraima and the epidemic grew exponentially in these Northern Brazilian states during the 1980s and 1990s, but its spreading rate seems to have slowed down since the early 2000s. The continuous molecular surveillance of major HIV-1 lineages spreading in Northern Brazilian will be crucial to understanding the HIV epidemic dynamics in this singular and changing region.

## Figures and Tables

**Table 1:** Human Immunodeficiency Virus Type 1 (HIV-1) *pol* (PR/RT) sequence dataset of non-pandemic subtype B variants (B_CAR_).

Clade	Location	Location Code	*N*	Sampling Time
B_CAR-BR-I_	Brazil/Amazonas	AM	45	2009–2017
	Brazil/Roraima	RR	29	2010–2017
	Brazil/Rondônia	RO	1	2015
	Brazil/São Paulo	SP	1	2003
B_CAR-SA-I_	Brazil/Amapá	AP	3	2013
	Brazil/Roraima	RR	7	2011–2013
	Brazil/Piauí	PI	1	2011
	French Guiana	GF	46	2006–2012
	Guyana	GY	7	2000–2013
	Suriname	SR	5	2000–2009
B_CAR-TT_	Trinidad and Tobago	TT	41	2000–2003
B_CAR-HISP_	Hispaniola	HISP	130	2003–2011

**Table 2:** Bayesian the most recent common ancestor (T_MRCA_) estimates for B_CAR_ clades from South America and the Caribbean.

Clade	T_MRCA_ Current Study	T_MRCA_ Ref. [5]	T_MRCA_ Ref. [6]	T_MRCA_ Ref. [2]
Subtype B	1970 (1963–1985)	-	1969 (1964–1974)	1966 (1962–1970)
B_CAR-TT_	1973 (1967–1980)	-	1973 (1970–1976)	1973 (1970–1976)
B_CAR-SA-I_	1976 (1971–1983)	1977 (1973–1981)	-	-
B_CAR-BR-I_	1979 (1974–1985)	-	1978 (1975–1981)	-

**Table 3:** Root location of the HIV-1 B_CAR-BR-I_ lineage.

Location	Complete Dataset (AM = 45 Sequences)	Subset 1 (AM = 22 Sequences)	Subset 2 (AM = 23 Sequences)
AM	92%	97%	93%
RR	1%	3%	6%
GF	0	0	1%

The table summarizes the posterior state probability distribution for the B_CAR-BR-I_ root location at the MCC trees obtained when the analyzed dataset included all the sequences originated in the states of Amazonas (*n* = 45), and when two random datasets containing near half of these sequences (*n* = 22 and 23) were used. AM: Amazonas); RR: Roraima GF: French Guiana.

## Supplementary Materials

The following are available online at https://www.mdpi.com/1999-4915/11/10/909/s1, Figure S1: Population evolution in the states of (A) Amazonas and (B) Roraima in the selected period of 1960 to 2010. Table S1: HIV-1 BCAR and subtype D pol sequences used for ML and Bayesian phylogenetic analyses. Table S2: Best fit demographic model for the HIV-1 BCAR-BR-I lineage.
