# Supplementary material for: Reconstructing the Dissemination Dynamics of the Major HIV-1 Subtype B Non-Pandemic Lineage Circulating in Brazil
Source: Viruses. 2019 Oct 1;11(10):909. doi: 10.3390/v11100909 (PMC6832740; doi:10.3390/v11100909)
Supplement: Supplementary file 1 [file viruses-11-00909-s001.pdf]

Supplementary Tables

Table S1: HIV-1 B_{CAR} and subtype D *pol* sequences used for ML and Bayesian phylogenetic analyses.

GenBank accession number	Location	Year
KU762067	BR AM	2010
KU762069	BR AM	2010
KU762080	BR AM	2010
KU762084	BR AM	2010
KU762087	BR AM	2010
KU762110	BR AM	2011
KU762111	BR AM	2011
KU762120	BR AM	2011
KU762125	BR AM	2011
KU762131	BR AM	2011
KU762134	BR AM	2011
KU762148	BR AM	2011
MH673114	BR AM	2011
MH673116	BR AM	2011
MH673118	BR AM	2011
MH673119	BR AM	2011
MH673072	BR AM	2012
MH673173	BR AM	2012
MH673175	BR AM	2012
MH673070	BR AM	2013
MH673074	BR AM	2013
MH673077	BR AM	2013
MH673084	BR AM	2013
MH673058	BR AM	2014
MH673087	BR AM	2014
MH673106	BR AM	2014
MH673156	BR AM	2014
MH673238	BR AM	2014
MH673130	BR AM	2015
MH673138	BR AM	2015
MH673143	BR AM	2015
MH673144	BR AM	2015
MH673153	BR AM	2015
MH673162	BR AM	2015
MH673156	BR AM	2015
MH673203	BR AM	2015
MH673221	BR AM	2015
MH673233	BR AM	2016
MH673234	BR AM	2016
MH673258	BR AM	2016
MH673265	BR AM	2016
MH673274	BR AM	2016
MH673277	BR AM	2016
MH673129	BR AM	2017
KU762273	BR RR	2010
KU762274	BR RR	2010
KU762279	BR RR	2010
KU762285	BR RR	2010
KU762287	BR RR	2011
KU762290	BR RR	2011
KU762293	BR RR	2011
KU762298	BR RR	2011
KU762299	BR RR	2011
KX443064	BR RR	2013

KX443082	BR RR	2013
KX443024	BR RR	2013
KX443028	BR RR	2013
KX443034	BR RR	2013
KX443035	BR RR	2013
KX443038	BR RR	2013
KX443044	BR RR	2013
KX443048	BR RR	2013
KX443049	BR RR	2013
KX443050	BR RR	2013
KX443057	BR RR	2013
KX443062	BR RR	2013
KX443063	BR RR	2013
KX443087	BR RR	2013
MH673194	BR RR	2015
MH673226	BR RR	2016
MH673247	BR RR	2016
MH673259	BR RR	2017
MH673195	BR RO	2015
JN692447	BR SP	2003
HQ127607	BR AM	2009
KT737294	BR AP	2013
KT737332	BR AP	2013
KT737333	BR AP	2013
KU762067	BR RR	2011
KU762069	BR RR	2011
KU762080	BR RR	2011
KU762084	BR RR	2013
KU762087	BR RR	2013
KU762110	BR RR	2013
KU762111	BR RR	2013
KU762120	BR RR	2013
KU762125	BR PI	2011
KT998279	GF	2012
KT998255	GF	2012
KT998261	GF	2012
KT998265	GF	2012
KT998266	GF	2012
KT998283	GF	2012
KT998218	GF	2011
KT998241	GF	2011
KT998251	GF	2011
KT998178	GF	2010
KT998179	GF	2010
KT998185	GF	2010
KT998186	GF	2010
KT998208	GF	2010
KT998209	GF	2010
KT998112	GF	2009
KT998118	GF	2009
KT998123	GF	2009
KT998145	GF	2009
KT998149	GF	2009
KT998150	GF	2009
KT998153	GF	2009
KT998156	GF	2009
KT998160	GF	2009
KT998163	GF	2009
KT998167	GF	2009

KT998055	GF	2008
KT998062	GF	2008
KT998076	GF	2008
KT998077	GF	2008
KT998081	GF	2008
KT998100	GF	2008
KT998109	GF	2008
KU052757	GF	2006
KU052754	GF	2006
KU052744	GF	2006
KT998021	GF	2007
KT998028	GF	2007
KT998030	GF	2007
KT998031	GF	2007
KT998033	GF	2007
KT998039	GF	2007
KT998045	GF	2007
KT998048	GF	2007
KT998051	GF	2007
KT998052	GF	2007
AY267325	SR	2000
KX390954	SR	2009
KX390958	SR	2009
KX390967	SR	2009
KX390888	SR	2009
AY267308	GY	2000
AY267309	GY	2000
AY267310	GY	2000
AY267311	GY	2000
AY267312	GY	2000
AY267313	GY	2000
DJSG100	GY	2013
EU439735	TT	2000
EU439738	TT	2003
AY267282	TT	2000
EU439753	TT	2000
EU439754	TT	2000
AY267273	TT	2000
AY267275	TT	2000
AY267289	TT	2000
AY267285	TT	2000
AY267277	TT	2000
AY267287	TT	2000
EU439734	TT	2000
AY267274	TT	2000
EU839606	TT	2000
AY267293	TT	2000
AY267283	TT	2000
AY267262	TT	2000
AY267279	TT	2000
EU439730	TT	2000
AY267271	TT	2000
AY267280	TT	2000
AY267259	TT	2000
EU439755	TT	2000
AY267292	TT	2000
EU439732	TT	2000
EU439742	TT	2000
EU439744	TT	2000

EU839610	TT	2001
EU439740	TT	2000
AY267281	TT	2000
EU439733	TT	2000
EU439739	TT	2003
AY267272	TT	2000
AY267278	TT	2000
EU439731	TT	2000
AY267291	TT	2000
EU439750	TT	2000
EU439756	TT	2000
AY267269	TT	2000
AY267270	TT	2000
AY267290	TT	2000
JN713675	DO	2010
JN713689	DO	2010
JN713625	DO	2008
EU439761	DO	2003
JN713673	DO	2009
EU439765	DO	2003
JN713615	DO	2008
JN713610	DO	2008
JN713707	DO	2008
JN713591	DO	2009
JN713677	DO	2009
EU439770	DO	2003
JN713569	DO	2009
JN713657	DO	2008
JN713680	DO	2008
JN713599	DO	2008
JN713602	DO	2009
EU439772	DO	2003
JN713572	DO	2009
JN713571	DO	2010
JN713630	DO	2007
JN713601	DO	2008
JN713598	DO	2007
JN713622	DO	2010
JN713668	DO	2007
JN713570	DO	2009
JN713577	DO	2010
KC340683	DO	2004
JN713616	DO	2009
JN713695	DO	2007
JN713613	DO	2009
JN713705	DO	2008
JN713581	DO	2010
JN713607	DO	2008
JN713635	DO	2008
JN713664	DO	2007
JN713573	DO	2008
JN713632	DO	2010
JN713688	DO	2007
JN713595	DO	2008
JN713701	DO	2009
JN713643	DO	2010
JN713579	DO	2008
JN713662	DO	2009
JN713708	DO	2007

EU439768	DO	2003
JN713600	DO	2008
JN713608	DO	2009
JN713642	DO	2007
JN713596	DO	2008
JN713678	DO	2009
KC340896	DO	2011
JN713655	DO	2010
JN713646	DO	2008
JN713656	DO	2009
JN713685	DO	2010
JN713611	DO	2007
JN713640	DO	2008
JN713605	DO	2007
JN713626	DO	2009
EU439764	DO	2003
EU439711	DO	2005
JN713621	DO	2008
JN713692	DO	2007
JN713690	DO	2009
JN713649	DO	2008
JN713647	DO	2010
JN713645	DO	2009
KC340442	DO	2008
JN713697	DO	2010
JN713676	DO	2008
JN713578	DO	2008
EU439769	DO	2003
JN713594	DO	2008
JN713612	DO	2008
JN713693	DO	2008
EU439762	DO	2003
JN713666	DO	2008
EU839598	DO	2005
JN713699	DO	2008
KC340840	DO	2010
JN713620	DO	2009
JN713703	DO	2009
JN713567	DO	2009
JN713574	DO	2009
JN713670	DO	2008
EU839597	DO	2005
JN713687	DO	2007
JN713644	DO	2009
JN713654	DO	2009
EU439766	DO	2003
JN713638	DO	2008
JN713585	DO	2008
JN713706	DO	2010
JN713609	DO	2007
JN713568	DO	2008
JN713652	DO	2007
JN713592	DO	2009
EU439763	DO	2003
JN713702	DO	2007
JN713587	DO	2008
JN713576	DO	2007
KC340780	DO	2008
EU439771	DO	2004

JN713661	DO	2008
JN713582	DO	2008
JN713629	DO	2010
JN713681	DO	2008
JN713709	DO	2010
KC340850	DO	2010
JN713633	DO	2008
JN713624	DO	2010
JN713669	DO	2009
JN713588	DO	2007
JN713631	DO	2007
EU439767	DO	2003
JN713665	DO	2008
JN713684	DO	2010
EU439719	HT	2005
EU439727	HT	2005
EU439725	HT	2005
EU439722	HT	2005
EU839602	HT	2005
EU839604	HT	2005
EU839600	HT	2005
EU839603	HT	2005
EU439721	HT	2005
EU839601	HT	2005
EU439718	HT	2004
EU439728	HT	2004
A34828	DRC	1983
A07108	DRC	1983
U88822	DRC	1984
M22639	DRC	1985
AJ287019	DRC	1997
AF357631	DRC	1998
AM041047	DRC	2002
AM041035	DRC	2002
KC340636	DRC	2005
FR666667	DRC	2007

DRC: Democratic Republic of Congo; HT: Haiti; DO: Dominican Republic; TT: Trinidad and Tobago; GF: French Guiana; GY: Guyana; SR: Suriname; BR: Brazil; AM: Amazonas; AP: Amapá; PI: Piauí; RR: Roraima; RO: Rondônia; SP: São Paulo.

Table S2. Best fit demographic model for the HIV-1 $B_{CAR\text{-}BR\text{-}I}$ lineage.

Clade	Model	GSS Log ml	Models compared	Log BF
$B_{CAR–BR\text{-}I}$	Log	-8676	-	-
	Expo	-8674	Log/Expo	
	Expa	-8681	Log/Expa	

Log marginal likelihood (ml) estimates for the logistic (Log), exponential (Expo) and expansion (Expa) growth demographic models obtained using the generalized stepping-stone sampling (GSS) method. The Log Bayes factor (BF) is the difference of the Log ml between alternative (H1) and null (H0) models (H1/H0). Log BFs > 3 indicates that model H1 is more strongly supported by the data than model H0.

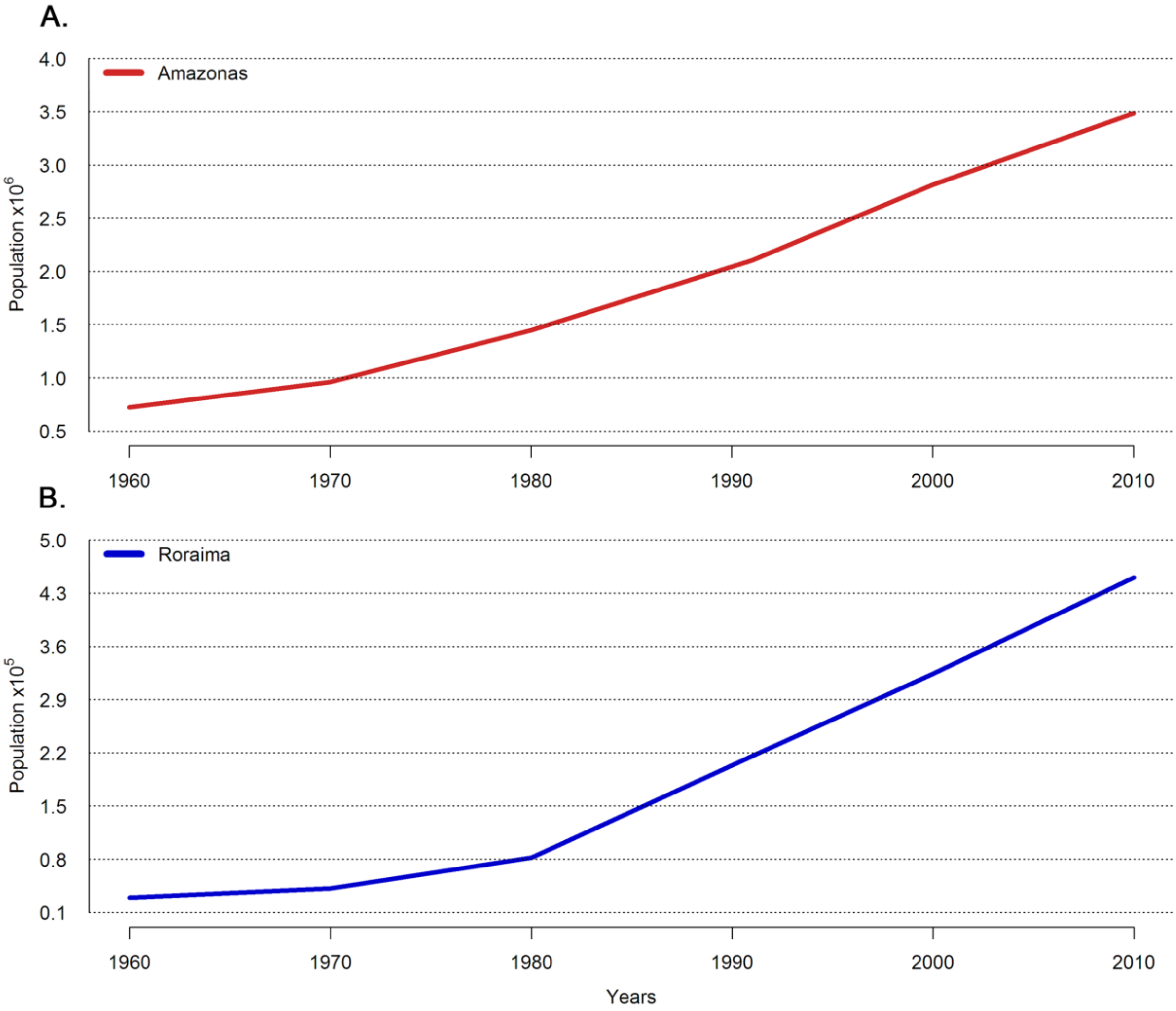

Figure S1. Population evolution in the states of (A) Amazonas and (B) Roraima in the selected period of 1960 to 2010.
